# Explicit meshfree ${{\varvec{u}}}-{{\varvec{p}}}_\mathbf{\mathrm{w}}$ solution of the dynamic Biot formulation at large strain

**DOI:** 10.1007/s40571-021-00436-8

**Published:** 2021-09-09

**Authors:** Pedro Navas, Miguel Molinos, Miguel M. Stickle, Diego Manzanal, Angel Yagüe, Manuel Pastor

**Affiliations:** 1 Department of Continuum Mechanics and Theory of Structures, Technical University of Madrid, Madrid, Spain; 2 Department of Mathematics Applied to Civil Engineering, Technical University of Madrid, Madrid, Spain

**Keywords:** Biot’s equations, Meshfree, Newmark predictor-corrector, Explicit approach, Large strains

## Abstract

In this paper, an efficient and robust methodology to simulate saturated soils subjected to low-medium frequency dynamic loadings under large deformation regime is presented. The coupling between solid and fluid phases is solved through the dynamic reduced formulation $u-p_\mathrm{w}$ (solid displacement – pore water pressure) of the Biot’s equations. The additional novelty lies in the employment of an explicit two-steps Newmark predictor-corrector time integration scheme that enables accurate solutions of related geomechanical problems at large strain without the usually high computational cost associated with the implicit counterparts. Shape functions based on the elegant Local Maximum Entropy approach, through the Optimal Transportation Meshfree framework, are considered to solve numerically different dynamic problems in fluid saturated porous media.

## Introduction

Modeling saturated soils under dynamic loads is of crucial importance when researchers deal with fast phenomena, like landslide propagation. Moreover, the more catastrophic 3D problems should also include large deformations. Despite the importance of both aspects, dynamic saturated problems and large deformations, research focused on these topics at once is limited. One of the main reasons why there is a lack of research in this paramount issue of geomechanics might be that a powerful and useful methodology requires complex hydro-mechanical models including inertial terms coupled with hyper-elastoplastic constitutive models where the deformation gradient acts as strain measure. Moreover, as analytical solutions can only be achieved for few idealized configurations, cutting edge numerical techniques must be considered to attain accurate and robust solutions in real-world problems.

The fluid saturated phenomenon has been widely studied in the numerical geotechnical field, where a big range of solutions can be found regarding the formulation considered for the coupled problem (either simplified or complete), the assumptions made with or without accelerations and the numerical techniques used to solve the equations, both in the spatial (mesh or meshfree-based techniques) and temporal dimension (explicit or implicit schemes).

The first formulations aimed to describe the physics behind a saturated porous medium are found in the governing equations introduced by Biot [4], later reviewed by Zienkiewicz and coworkers [53–56]. Similar equations were obtained by Lewis and Schrefler [20] within the Hybrid Mixture Theory, in this case, starting from the microscopic scale, improving the consistency and robustness of the formulation. Regarding the inertial terms, both acceleration of fluid and solid phases are employed in the complete formulation, covering a wide range of frequencies [18, 39]. This formulation is usually expressed in terms of the relative water displacements, *w*, which has been proved to be successful [24, 29]. However, other research presents this complete formulation by means of the total displacement of the water, *U*, as a nodal unknown (Ye et al. [49] and Sladek et al. [46]). Concerning the simplified formulations, the $u-w$ approach is computationally more expensive than the $u-p_\mathrm{w}$ since the former employs more degrees of freedom per node. Thus, its utilization is not recommended when the $u-p_\mathrm{w}$ formulation is sufficient to capture the complete wave propagation in a saturated soil problem. The $u-p_\mathrm{w}$ (solid displacement–fluid pressure) formulation is widely used in dynamics to solve different hydromechanical coupled problems due to its simplicity as well as the high accuracy achieved for a great variety of geomechanical problems (e.g., [10, 53, 54]) .

All these formulations have been usually solved in time through an implicit scheme [1, 6, 8, 15, 34]. Recently, Navas et al. proposed an explicit solution of the $u-w$ formulation with excellent results, see [33]. Explicit schemes are a feasible alternative in which there is no need to compute the tangent stiffness matrix, avoiding the complex linearization of the governing equations. Moreover, the computational effort is minimized as forward values are computed directly from the current one, avoiding the solution of nonlinear equations system when advancing in time. Finally, explicit schemes allow a more efficient use of multi-core processor, thus facilitating a parallel programming paradigm.

Regarding the application of the Biot’s equations under large deformation regime, the first works were carried out by Diebels and Ehlers [14], Borja et al. [6, 8] and Armero [1] who tested their models by simulating the constitutive behavior of the the solid phases with an hyperelastic, Cam-Clay and Drucker–Prager theories, respectively. Around the same period, Ehlers and Eipper [15] applied a new Neo-Hookean constitutive model to represent the compaction of the soil up to the solid compaction point. An interesting extension was made by Sanavia et al. to unsaturated soils [42–44]. Again, most of these models were solved employing implicit schemes where the linearization of the $u-p_\mathrm{w}$ equations was necessary. There is a scarcity of examples in the specialized literature of explicit solutions in time for the $u-p_\mathrm{w}$ formulation under large deformation regime. However, for saturated porous media undergoing a fast deformation process, this type of time integration schemes is a feasible alternative, as the usual restriction required for the time step to attain a stable solution can be assumed, as far as numerical efficiency is concerned. The present research aims to cover the lack of explicit time integration solutions for the $u-p_\mathrm{w}$ formulation undergoing large deformations.

In recent years, in the computational mechanics field, large strain approaches go hand in hand with meshfree methods due to their numerous advantage to reproduce large relative displacements. In the geotechnical field, this combination of tools shows excellent results in problems such as landslides, liquefaction or other natural disasters. Saturated soils are also modeled through these approaches. Recent promising works can be found in the literature like Pastor et al. [38] with the Smooth Particle Hydrodynamics (SPH) and the works of Bandara and Soga [3], Ceccato and Simonini [11] or Zhao and Choo [51] with the Material Point Method (MPM). Precisely, with this meshfree scheme, we find excellent contributions to the explicit $u-p_\mathrm{w}$ approach (see [50, 52]). The small strain approach is employed within this research.

The goal of the present research is the proposal of a robust predictor-corrector explicit algorithm for the $u-p_\mathrm{w}$ formulation at large strain where the spatial domain has been discretized into nodes and material points following the Optimal Transportation Meshfree (OTM) scheme of Li et al. [21]. The shape functions developed by Arroyo and Ortiz [2] based on the principle of maximum entropy [37] are also employed.

The rest of the paper is organized as follows. The Biot’s equations are presented in Sect. 2, with emphasis within the $u-p_\mathrm{w}$ formulation. The constitutive models employed to model the solid behavior are summarized in Sect. 3. The discretization techniques, highlighting the meshfree solution and the explicit methodology, are provided in Sect. 4. Applications to various problems are illustrated in Sect. 5. Relevant conclusions and future lines are drawn in Sect. 6. The definitions of all symbols used in the equations are provided in the nomenclature appendix.

## Biot’s equations: $u-p_\mathrm{w}$ formulation

The Biot’s equations [4] are based on formulating the mechanical behavior of a solid-fluid mixture, the coupling between different phases, and the continuity of flux through a differential domain of saturated porous media. Hereinafter, the balance equations will be derived from Lewis and Schrefler [20] in the spatial setting (see [20] or [43, 44] for the kinematic equations), departing from the more general equation, and, in order to reach the compact $u-p_\mathrm{w}$ form, making the necessary hypotheses.

Concerning the notation, bold symbols are employed herein for vectors and matrices as well as regular letters for scalar variables. Let $\varvec{u}$ and $\varvec{U}$ represent the displacement vector of the solid skeleton and the absolute displacement of the fluid phase, respectively. Since in porous media theory is common to describe the fluid motion with respect to the solid, the relative displacement of the fluid phase with respect to the solid one, $\varvec{w}$, is introduced and expressed as [25]

1 $$\begin{aligned} \varvec{ w }=n S_\mathrm{w}\, \varvec{ \left( U-u\right) }, \end{aligned}$$

where $S_\mathrm{w}$ is the degree of water saturation and *n* the soil porosity. Note that $\varvec{ \left( U-u\right) }$ is usually termed as $\varvec{u}^{ws}$ in the literature [20].

Let $\rho$, $\rho _{w}$ and $\rho _{s}$, respectively, represent the mixture, fluid phase, and solid particle densities, the mixture density can be defined as function of the porosity:

2 $$\begin{aligned} \rho =n S_\mathrm{w} \rho _\mathrm{w}+(1-n) \rho _\mathrm{s}. \end{aligned}$$

In the above equations, the porosity, *n*, is the ratio between the voids volume, $V_\mathrm{v}$, and the total volume, $V_\mathrm{T}$:

3 $$\begin{aligned} n=\frac{V_\mathrm{v}}{V_\mathrm{T}}=\frac{V_\mathrm{v}}{V_\mathrm{v}V_\mathrm{s}}, \end{aligned}$$

where $V_s$ is the volume of the solid grains.

Next, we first explain in detail the derivation of mass balance and linear momentum equations for a fluid saturated multiphase media. Then, the final $u-p_\mathrm{w}$ formulation is presented. The following equations are first given by Lewis and Schrefler [20]. In this research, $D^s/Dt$ denotes the material time derivative with respect to the solid, considering:

$$\begin{aligned} \varvec{a}^s = \ddot{\varvec{u}} = \frac{D^s \dot{\varvec{u}}}{Dt} = \frac{D^{2s} \varvec{u}}{Dt^2}\\ n S_\mathrm{w} \varvec{a}^{ws} = \ddot{\varvec{w}} = \frac{D^s \dot{\varvec{w}}}{Dt} = \frac{D^{2s} \varvec{w}}{Dt^2} \end{aligned}$$

where $\varvec{a}^s$ and $\varvec{a}^{ws}$ are the solid acceleration and the relative water acceleration with respect to the solid, respectively, being the proposed expressions based on the relationships $\varvec{\dot{u}} \equiv \varvec{v}^{s}$ and $\varvec{\dot{w}} \approx nS_\mathrm{w} \varvec{v}^{ws}$.

### Derivation of the mass balance equation

The general mass balance equation in a multiphase media for compressible grains is presented next. Let $p_\mathrm{w}$, $p_\mathrm{g}$ represent the water and gas pressures, respectively, *T*, the temperature, then this general mass balance equation is written as follows,

4 $$\begin{aligned}&\Big ( \frac{\alpha -n}{K_\mathrm{s}}S_\mathrm{w}^2+\frac{nS_\mathrm{w}}{K_\mathrm{w}}\Big )\frac{D^s p_\mathrm{w}}{D t} + \frac{\alpha -n}{K_\mathrm{s}}S_wS_g\frac{D^s p_\mathrm{g}}{D t} \nonumber \\&\quad -\beta _\mathrm{sw}\frac{D^s T}{D t} + \left( \frac{\alpha -n}{K_\mathrm{s}}S_\mathrm{w} p_\mathrm{w}-\frac{\alpha -n}{K_\mathrm{s}}S_\mathrm{w} p_\mathrm{g}+n\right) \frac{D^s S_\mathrm{w}}{D t} \nonumber \\&\quad + \alpha S_\mathrm{w} \text{ div } \dot{\varvec{u}} + \frac{1}{\rho _\mathrm{w}} \text{ div } (\rho _\mathrm{w} \dot{\varvec{w}}) = - n e^w, \end{aligned}$$

where the right hand side term represents the quantity of water lost through evaporation for unit time and volume. The thermal expansion coefficient of the solid-fluid mixture, $\beta _\mathrm{sw}$, is a combination of that of the solid, $\beta _\mathrm{s}$, and the fluid, $\beta _\mathrm{w}$:

5 $$\begin{aligned} \beta _\mathrm{sw} = S_\mathrm{w} [(\alpha -n)\beta _\mathrm{s} +n \beta _\mathrm{w}]. \end{aligned}$$

In addition, $\alpha$ is the Biot’s coefficient:

6 $$\begin{aligned} \alpha =1-\frac{K}{K_\mathrm{s}}. \end{aligned}$$

where *K* denotes the bulk modulus of the solid skeleton. Biot’s coefficient may be usually assumed equal to one in soils as the grains are less deformable than the mixture.

In the current work, the soil is assumed to be totally saturated, i.e., $V_\mathrm{v}$ coincides with the water volume, which results $S_\mathrm{w}$ equals to one and $S_g=0$. As we also consider iso-thermal multiphase media, $D^s T/D t =0$, $e^w=0$, consequently, $D^s S_w/D t =0$. Taking into account all these assumptions, the volumetric compressibility of the mixture, *Q* [54], can be calculated as

7 $$\begin{aligned} Q = \left[ \frac{1-n}{K_s} + \frac{n}{K_\mathrm{w}} \right] ^{-1}, \end{aligned}$$

where $K_s$ is the bulk modulus of the solid grains, whereas $K_w$ is the compressive modulus of the fluid phase (usually water).

If additionally the spatial variation of the fluid density is neglected and we take into consideration Eq. (7), (4) is simplified as,

8 $$\begin{aligned} \frac{\dot{p_\mathrm{w}}}{Q} + \text{ div } \varvec{\dot{u}} + \text{ div } \varvec{\dot{w}} = 0 , \end{aligned}$$

### Linear momentum balance equations

On the one hand, the relative velocity of the fluid, $\dot{\varvec{w}}$, in Eq. (4) is defined through the generalized Darcy law as [20]

9 $$\begin{aligned} \dot{\varvec{w}}=\frac{k^{rw}\varvec{k}}{\mu _\mathrm{w}}\left[ -\text{ grad } \,p_\mathrm{w} + \rho _\mathrm{w}(\varvec{g}-\ddot{\varvec{u}}-\frac{\ddot{\varvec{u}}}{n})\right] , \end{aligned}$$

where $\varvec{g}$ represents the gravity acceleration vector, $\varvec{k}$, the intrinsic permeability tensor of the porous matrix in water saturated condition, considered isotropic in this research ($\varvec{k}=k \varvec{I}$), $k^{rw}$ is the water relative permeability parameter (a dimensionless parameter varying from zero to one) and $\mu _\mathrm{w}$ is the dynamic viscosity of the water [Pa $\cdot$ s]. The *intrinsic* permeability *k*, expressed in [m$^{2}$], is related with the notion of hydraulic conductivity, $\kappa$ [m/s], by the following equation

10 $$\begin{aligned} \frac{k}{\mu _\mathrm{w}}=\frac{\kappa }{\rho _\mathrm{w} g}. \end{aligned}$$

On the other hand, according to Lewis and Schrefler [20], the linear momentum balance equation for the multiphase system can also be expressed as the summation of the dynamic equations for the individual constituents relative to the solid as, i.e.,

11 $$\begin{aligned} -\rho \ddot{\varvec{u}} - \rho _\mathrm{w}\ddot{\varvec{w}} + \text{ div } \varvec{\sigma }+\rho \varvec{g}=\varvec{0}, \end{aligned}$$

where the convective terms, related to the acceleration terms, have been neglected, which is normal in soils.

In the calculation of the internal forces of the soil, the Terzaghi’s effective stress theory [47] will be followed, which is defined as follows:

12 $$\begin{aligned} \varvec{ \sigma } =\varvec{ \sigma '} - p_\mathrm{w}{} \mathbf{I} , \end{aligned}$$

where $\varvec{ \sigma '}$ and $\varvec{ \sigma }$ are the respective effective and total Cauchy stress tensors (positive in tension), whereas $\mathbf{I}$ is the second-order unit tensor. Contrary, pore pressure $p_\mathrm{w}$ is assumed positive for compression.

Plugging Eq. (12) into Eq. (11), the linear momentum equation can be written as follows

13 $$\begin{aligned} \text{ div } \left[ \varvec{ \sigma '} - p_{w} \, \mathbf{I} \right] -\rho \varvec{\ddot{u}}-\rho _\mathrm{w}\varvec{\ddot{w}}+\rho \varvec{g}=\varvec{0}. \end{aligned}$$

### The $u-p_\mathrm{w}$ formulation

Considering the three Biot’s equations, the $\varvec{u}-p_\mathrm{w}$ assumes that accelerations of the fluid phase are negligible. Thus, Eq. (13) yields:

14 $$\begin{aligned} \text{ div } \left[ \varvec{ \sigma '} - p_\mathrm{w} \, \mathbf{I} \right] -\rho \varvec{\ddot{u}}+\rho \varvec{g}=\varvec{0}. \end{aligned}$$

Moreover, in order to avoid the employment of $\varvec{w}$ as a degree of freedom of our problem, Eqs. (8) and (9) can be combined and the mass equation can be expressed as

15 $$\begin{aligned} \dot{p_\mathrm{w}} = -Q\left[ \text{ div } \dot{\varvec{u}} + \frac{\varvec{k}}{\mu _\mathrm{w}} \text{ div }\left( \rho _\mathrm{w} \varvec{g} - \rho _\mathrm{w} \ddot{\varvec{u}} - \text{ grad } p_\mathrm{w}\right) \right] .\nonumber \\ \end{aligned}$$

## Constitutive models for the solid phase

In this Section, the hyperelastic and hyper-elastoplastic models, employed within this research, are outlined. Further information of both constitutive laws can be found in [30, 33, 34].

### Neo-Hookean material model extended to compressible range

In this research, the Neo-Hookean constitutive behavior has been considered as a extension of the elastic one in the large strain regime. Moreover, among several variants, the one proposed by Ehlers and Eipper [15] has been chosen. This law takes into consideration the compaction point of the soil, from the influence of the initial porosity $n_0$ and the Jacobian, calculated as the determinant of the deformation gradient $\varvec{F}$, in the following manner:

16 $$\begin{aligned} \varvec{\tau }'=G(\varvec{b}-\mathbf{I} )+\lambda \, n_0^2\left( \frac{J}{n_0}-\frac{J}{J-1+n_0} \right) \mathbf{I} , \end{aligned}$$

where $\varvec{\tau }'$ and $\varvec{b}$ are the effective Kirchhoff stress tensor and the left Cauchy–Green tensor, respectively, whereas *G* and $\lambda$ are the Lamé constants.

### Drucker–Prager yield criterion

In order to reproduce frictional-cohesive behavior at large strain, the traditional Drucker–Prager yield criterion [41, 44] has been extended to large strain procedure. This methodology follows the work of Ortiz, Simo and coworkers [12, 36, 45] to relate the left Cauchy–Green strain tensor $\mathbf {b}$, calculated at the current configuration, and the small strain tensor $\varvec{\varepsilon }$. Indeed, for the current loading step, $k+1$, the trial elastic deformations, pressure ($p^{trial}_{k+1}$), and the deviatoric stress tensor ($\mathbf {s}^{trial}_{k+1}$) are computed as the elastic deformations, pressure and the deviatoric stress tensor are computed as:

17 $$\begin{aligned} \mathbf {b}^{e\;\mathrm{trial}}_{k+1}= & {} \Delta \mathbf {F}_{k+1}\mathbf {b}^e_{k+1}(\Delta \mathbf {F}_{k})^{T}, \end{aligned}$$

18 $$\begin{aligned} \varvec{\varepsilon }^{e\; \mathrm{trial}}_{k+1}= & {} \frac{1}{2}\,\log \mathbf {b}^{e\; \mathrm{trial}}_{k+1}, \end{aligned}$$

19 $$\begin{aligned} p^\mathrm{trial}_{k+1}= & {} K \left( \varepsilon ^{e}_{vol}\right) ^\mathrm{trial}_{k+1}, \end{aligned}$$

20 $$\begin{aligned} \mathbf {s}^\mathrm{trial}_{k+1}= & {} 2G\,\left( \varvec{\varepsilon }^{e}_{dev}\right) ^\mathrm{trial}_{k+1}. \end{aligned}$$

where K and G represent the bulk and shear moduli of the solid, respectively. $\Delta \mathbf {F}_{k+1}$ is the incremental form of the deformation gradient, calculated as:

21 $$\begin{aligned} \Delta \mathbf {F}_{k+1} = \mathbf {I}+\sum _{a=1}^{Nb}\Delta u_{k+1}^a \otimes \nabla N^a(x_{k}^p), \end{aligned}$$

where $\nabla N^a$ is the gradient of the shape function, in this case, the Local Max-Ent, defined in Sect. 4.1.

Regarding the Drucker–Prager yield criterion, the employed methodology allows to distinguish if the location of the stress state is on the cone or apex before calculating the plastic strain. The yield conditions for the classical and apex regions, respectively, are:

22 $$\begin{aligned} \Phi ^{cl}= & {} \Vert \mathbf{s} ^\mathrm{trial}_{k+1}\Vert - 2G\Delta \gamma + 3\alpha _{_F}[p^\mathrm{trial}_{k+1}- 3K\alpha _{_Q}\Delta \gamma ] \nonumber \\&-\beta c_{k+1}, \end{aligned}$$

23 $$\begin{aligned} \Phi ^{ap}= & {} \frac{\beta }{3\alpha _{_F}}\left[ c_k+H\sqrt{\Delta \gamma _1^2 + 3\alpha _{_Q}^2(\Delta \gamma _1+\Delta \gamma _2)^2} \right] \nonumber \\&- p^\mathrm{trial}_{k+1} +3K\alpha _{_Q}\left( \Delta \gamma _1+\Delta \gamma _2 \right) , \end{aligned}$$

where $\Delta \gamma _1=\frac{\Vert \mathbf{s} ^\mathrm{trial}_{k+1}\Vert }{2G}$, $\Delta \gamma$ and $\Delta \gamma _2$ are the objective functions to be calculated in the Newton–Raphson scheme for the classical or apex regions accordingly. $c_k$ is the cohesion of the material, *H* the hardening parameter and $\alpha _F$,$\alpha _Q$ and $\beta$ are material parameters that depend on friction and dilatancy angles as well as the shape of the yield surface, taking into account that the Drucker–Prager criterion employs a cone to approximate the Mohr–Coulomb surface and this cone can be outer or inner to the aforementioned surface (more information is found in [33]).

A limit value for the pressure, $p_\mathrm{lim}$, is necessary to know which algorithm is to be employed. If the trial pressure is lower than this limit, classical return-mapping algorithm is employed, being this limit written as:

24 $$\begin{aligned} p_\mathrm{lim}= & {} \frac{3\alpha _{_Q}K}{2G}\Vert \mathbf{s} ^\mathrm{trial}_{k+1}\Vert \nonumber \\&+\frac{\beta }{3\alpha _{_F}}\left( \frac{\Vert \mathbf{s} ^\mathrm{trial}_{k+1}\Vert }{2G}H\sqrt{1+3\alpha _{_Q}^2} + c_k \right) . \end{aligned}$$

The equivalent plastic strain, ${\overline{\varepsilon }}^p_{k+1}$, is calculated in different ways depending on the stress state, whether it is in the classical or in the apex region:

$$\begin{aligned} {\overline{\varepsilon }}^p_{k+1}={\overline{\varepsilon }}^p_{k}+\Delta \gamma \sqrt{3\alpha _{_Q}^2+1}\\ {\overline{\varepsilon }}^p_{k+1}={\overline{\varepsilon }}^p_{k}+\sqrt{\Delta \gamma _1^2+3\alpha _{_Q}^2\left( \Delta \gamma _1+\Delta \gamma _2 \right) ^2} \end{aligned}$$

## Discretization of the solution: explicit scheme

To solve the aforementioned coupled problem in the time domain, the standard central difference explicit Newmark time integration scheme is employed. If the current time step is numbered as $k+1$, and assuming the solution in the previous step *k* has been already obtained (hence it is known), a relationship between $\mathbf{u} _{k+1}$, $\dot{\mathbf{u }}_{k+1}$ and $\ddot{\mathbf{u }}_{k+1}$ is established according to a finite difference scheme, as follows:

25 $$\begin{aligned} \ddot{\varvec{u}}_{k+1}= & {} \ddot{\varvec{u}}_{k}+\Delta \ddot{\varvec{u}}_{k+1}, \nonumber \\ \dot{\varvec{u}}_{k+1}= & {} \dot{\varvec{u}}_k+\ddot{\varvec{u}}_{k}\Delta t+\gamma \Delta t \Delta \ddot{\varvec{u}}_{k+1}, \nonumber \\ \varvec{u}_{k+1}= & {} \varvec{u}_{k}+\dot{\varvec{u}}_{k} \Delta t+\frac{1}{2} \Delta t^{2} \ddot{\varvec{u}}_{k}+\beta \Delta t^{2}\Delta \ddot{\varvec{u}}_{k+1}. \end{aligned}$$

Similarly, the pore pressure, evaluated at material point level, can be expressed in terms of its derivative.

26 $$\begin{aligned} p_{\mathrm{w}_{k+1}}= & {} p_{\mathrm{w}_k}+\dot{p}_{\mathrm{w}_k}\Delta t+\theta \Delta t \Delta \dot{p}_\mathrm{w}{_{k+1}}. \end{aligned}$$

When the Newmark scheme parameters, $\gamma$ and $\beta$, are set to 0.5 and 0, respectively, the central difference scheme is obtained. In the present research, $\theta =\gamma =0.5$. Rearranging terms, *Predictor* and *Corrector* terms can be obtained:

27 $$\begin{aligned} \dot{u}_{k+1}= & {} \underline{\dot{u}_{k}+(1-\gamma )\Delta t \, \ddot{u}_{k}} + \gamma \Delta t \, \ddot{u}_{k+1} , \\ p_{\mathrm{w}_{k+1}}= & {} \underline{p_{\mathrm{w}_{k}}+(1-\gamma )\Delta t \, \dot{p_\mathrm{w}}_{k}} + \gamma \Delta t \, \dot{p_\mathrm{w}}_{k+1} ; \end{aligned}$$

being the underlined terms the ones of the predictor step, which will be called $\dot{u}_{k+*}$ and $p_{\mathrm{w}_{k+*}}$. For further details, the reader is referred to Sect. 4.2.1.

About the numerical stability of the proposed methodology, it is guaranteed when the Courant–Friedrichs–Lewy (CFL) condition is satisfied. In particular, the time step, $\Delta t$, should be small enough to ensure that the compressive wave can travel between nodes, i.e.,

28 $$\begin{aligned} \Delta t < \frac{\Delta x}{V_c}, \end{aligned}$$

where $\Delta x$ represents the minimum mesh size, further details about the procurement of this parameter will be given in the following subsection. Finally, $V_\mathrm{c}$ is the *p*-wave velocity (see [55]), which is defined by

29 $$\begin{aligned} V_c=\sqrt{\left( D+\frac{K_\mathrm{w}}{n}\right) \frac{1}{\rho }}, \; \; \text {where} \;\; D=\frac{2G(1-\nu )}{1-2\nu }. \end{aligned}$$

### Spatial discretization

The Optimal Transportation Meshfree [17, 21, 22] has been demonstrated to perform reasonably well in geotechnical problems and, specifically, in multiphase problems [31]. It is based in the conjunction of material points and nodes. As mentioned before, the shape functions are based on the work of Arroyo and Ortiz [2], who defined the Local Max-Ent shape function (LME) of the material point $\varvec{(}x)$ with respect to the neighborhood $\varvec{(}x_a)$ as follows:

30 $$\begin{aligned} N_a(\mathbf{x} )=\frac{\exp \left[ -\beta _{_\mathrm{LME}} \; |\mathbf{x}-x_\mathrm{a}|^2 + \varvec{\lambda }^* \cdot (x-x_{a}) \right] }{Z(\mathbf{x} ,\varvec{\lambda }^*(\mathbf{x} ))}, \end{aligned}$$

where the computation is done along a neighborhood $N_b$ and

31 $$\begin{aligned} Z(\mathbf{x}, {\varvec{\lambda }}) = \sum _{a=1}^{Nb}{ \exp \left[ -\beta _{_{LME}} \, |\mathbf{x-x_{a}}|^2 + \varvec{\lambda } \cdot \mathbf {(x-x_{a})} \right] }. \end{aligned}$$

The first derivatives of the shape function can be obtained from the own shape function and its Hessian matrix **J** by employing the following expression:

32 $$\begin{aligned} \nabla N^*_a= & {} -N^*_a \, (\mathbf{J} ^*)^{-1} \, (\mathbf{x}-x_a) , \end{aligned}$$

The parameter $\beta _{_\mathrm{LME}}$ defines the shape of the neighborhood, and it is related with the discretization size (or nodal spacing), *h*, and the constant, $\gamma _{_\mathrm{LME}}$, which controls the locality of the shape functions, as follows,

33 $$\begin{aligned} \beta _{_\mathrm{LME}}=\frac{\gamma _{_\mathrm{LME}}}{h^2}. \end{aligned}$$

It bears emphasis that $\varvec{\lambda }^*(\mathbf{x})$ comes from the minimization of the function $g(\varvec{\lambda })=\log Z(\mathbf{x}, \varvec{\lambda })$ to guarantee the maximum entropy. Moreover, in the remapping of the shape function, before recomputing the aforementioned minimization process, it is necessary to update the neighborhood and the parameter $\beta _{_{\mathrm{LME},k+1}}^p < \beta _{_{\mathrm{LME},k}}^p$ in order to improve the stability.

The discretization size, *h*, is an interesting topic when dealing with explicit schemes in the OTM methodology. Although the neighborhood or influence radius is larger than in the traditional FEM [21], we should take *h* as the distance between the current node and the closest one since it is the more limiting one. Furthermore, the value of $\Delta x$ expressed in Eq. 29 will be obtained as the minimum value of *h* for each node in the whole nodal set.

By employing the outlined shape functions and applying Galerkin procedure to the weak form of Eqs. (11) and (13) (See [41, 44] for details), the following matrix equations appear:

34 $$\begin{aligned} \varvec{R}^s- \varvec{R}^w- \varvec{M}^s \ddot{\varvec{u}} + \varvec{f}^{\mathrm{ext}, \, s}= & {} \varvec{0} \end{aligned}$$

35 $$\begin{aligned} - \varvec{C} \dot{\varvec{u}} + \varvec{M}^w \ddot{\varvec{u}} + \varvec{f}^{\mathrm{ext}, \, w}-\varvec{R}^{w}= & {} \dot{p_\mathrm{w}} \end{aligned}$$

where the internal and external forces are defined as:

36 $$\begin{aligned} \varvec{R}^s= & {} \sum _{P=1}^{N_{P}} V_{P} \varvec{\sigma '} \nabla \mathbf {N} \nonumber \\ \varvec{R}^w= & {} \sum _{P=1}^{N_{P}} V_{P} p_\mathrm{w} \nabla \mathbf {N} \nonumber \\ \varvec{f}^{\mathrm{ext}, \, s}= & {} \varvec{M}^s \varvec{g} - \int _{\partial \Omega _{\tau }} \varvec{\sigma '} \varvec{n}\mathbf {N} d \Gamma \nonumber \\ \varvec{f}^{\mathrm{ext}, \, w}= & {} \varvec{M}^w \varvec{g} + \int _{\partial \Omega _{p_\mathrm{w}}} p_\mathrm{w} \varvec{n} \mathbf {N} d \Gamma , \nonumber \end{aligned}$$

and the mass and damping matrices, constructed as lumped matrices in order to alleviate the computational effort of the explicit scheme, are written as follows:

$$\begin{aligned} \varvec{M}^s= & {} \sum _{P=1}^{N_{P}} V_{P} \rho \mathbf {N} \\ \varvec{M}^w= & {} \sum _{P=1}^{N_{P}} Q \rho _\mathrm{w} \frac{k}{\mu _\mathrm{w}} V_{P} \mathbf {B}\mathbf {m} \mathbf {N} \\ \varvec{C}^w= & {} \sum _{P=1}^{N_{P}} Q V_{P} \mathbf {B}\mathbf {m} \mathbf {N} \end{aligned}$$

being $V_p$ and $N_p$ the volume and the neighborhood of a material point P, respectively, $\mathbf {B}$ the symmetric shape function gradient operator and $\mathbf {m}$ the identity matrix in Voigt notation. Thus, $\mathbf {B}\mathbf {m}$ reproduces the *divergence* operation.

### Explicit integration

The proposed scheme seeks the value of the solid acceleration, $\ddot{\varvec{u}}$, calculated from Eq. (35). It is worth mentioning that the subscript $k+1$ is employed for the current step and *k* in the previous one. Furthermore, in this calculation, it is necessary to predict the internal forces from the values of the predicted solid displacement, $\varvec{u}_{k+*}$, and the predicted pore pressure, $p_{w_{k+*}}$. The stress has to be calculated in this predicted step as well:

$$\begin{aligned} \varvec{\sigma '}_{k+*}=\varvec{\sigma '}(\varvec{F}_{k+*})=\varvec{\sigma '}(\varvec{F}(\varvec{u}_{k+*})) \end{aligned}$$

Moreover, the approximation of the logarithmic strain as the measure to be employed in the deformed configuration has been demonstrated to provide good performance when large deformations are modeled (see [7, 9, 44]). In the present research, the tensor $\mathbf {b}$, the Left Cauchy–Green strain tensor ($\mathbf {b}=\mathbf {F}\mathbf {F}^T$) depends on the displacement on the predicted step as follows:

$$\begin{aligned} \mathbf {b}_{k+*}=\mathbf {b}(\mathbf {F}_{k+*})=\mathbf {b}(\mathbf {F}(\varvec{u}_{k+*})) \end{aligned}$$

Once the solid acceleration is reached, the pore pressure velocity can be calculated from Eq. (36). Also, in this equation, water internal forces and solid velocities have to be evaluated in the predicted step, $k+*$.

All these ingredients are those which integrate the Newmark Predictor-Corrector explicit algorithm for the $\varvec{u}-p_\mathrm{w}$ formulation at large strain. Its numerical implementation is explained in the following section.

#### Explicit algorithm within the OTM framework

The pseudo-algorithm of the whole model can be written as follows. The employment of the superscript *p* for material point calculations has to be pointed out.

1. Explicit Newmark Predictor ($\gamma =0.5$, $\beta =0$)
  $$\begin{aligned} u_{k+1}= & {} u_k+\Delta t \dot{u}_{k}+0.5\Delta t^2 \, \ddot{u}_k, \\ \dot{u}_{k+*}= & {} \dot{u}_{k}+(1-\gamma )\Delta t \, \ddot{u}_{k}, \\ p_{w_{k+*}}= & {} p_{w_k}+(1-\gamma )\Delta t \, \dot{p}_{w_k}. \end{aligned}$$
2. Nodes and Material points position update
  $$\begin{aligned}&x_{k+1}=x_{k}+\Delta u_{k+1},\\&x_{k+1}^p=x_{k}^p+\sum _{a=1}^{Nb}\Delta u_{k+1}^a \otimes N^a(x^p_{k}). \end{aligned}$$
3. Deformation gradient calculation and related parameters
  $$\begin{aligned} \mathbf {F}_{k+1}= & {} \Delta \mathbf {F}_{k+1} \mathbf {F}_{k}, \\ V= & {} JV_0=\det \mathbf {F} \, V_0,\\ n= & {} 1-\frac{1-n_0}{J}. \end{aligned}$$
4. Update density and recompute lumped mass
  $$\begin{aligned} \rho _{k+1}=n_{k+1}\rho _\mathrm{w}+(1-n_{k+1})\rho _s. \end{aligned}$$
5. Remapping loop, reconnect the nodes with their new material neighbors.
6. Constitutive relations from the Elasto-Plastic model, $\varvec{\sigma '}_{k+*}$ and internal forces $\varvec{R}^s_{k+*}$ and $\varvec{R}^w_{k+*}$.
7. Calculate $\varvec{\ddot{u}}_{k+1}$ from Eq. (35):
  $$\begin{aligned} \ddot{\varvec{u}}_{k+1} = \left[ \varvec{M}^s \right] ^{-1}\left[ \varvec{R}^s_{k+*}- \varvec{R}^w_{k+*} + \varvec{f}^{ext, \, s}_{k+1}\right] \end{aligned}$$
8. Calculate $\dot{p_\mathrm{w}}_{k+1}$ from Eq. (36):
  $$\begin{aligned} \dot{p_\mathrm{w}}_{k+1}=- \varvec{C} \dot{\varvec{u}}_{k+*} + \varvec{M}^w \ddot{\varvec{u}}_{k+1} + \varvec{f}^{\mathrm{ext}, \, w}_{k+1}-\varvec{R}^\mathrm{w}_{k+*} \end{aligned}$$
9. Explicit Newmark Corrector
  $$\begin{aligned}&\dot{u}_{k+1}=\dot{u}_{k+*}+\gamma \Delta t \, \ddot{u}_{k+1},\\&{p}_{\mathrm{w}_{k+1}}={p}_{\mathrm{w}_{k+*}}+\gamma \Delta t \, \dot{p}_{\mathrm{w}_{k+1}}. \end{aligned}$$

## Verification examples

This section is composed by two different problems. The first one deals with a consolidation, either pseudo-static or cyclic one, in order to validate the model in typical porous media applications. The second one, seeking the assessment of the performance of the proposed algorithm in a real geotechnical problem, studies the failure of a vertical wall of saturated soil.

### Consolidation of a column of soil

**Fig. 1:**
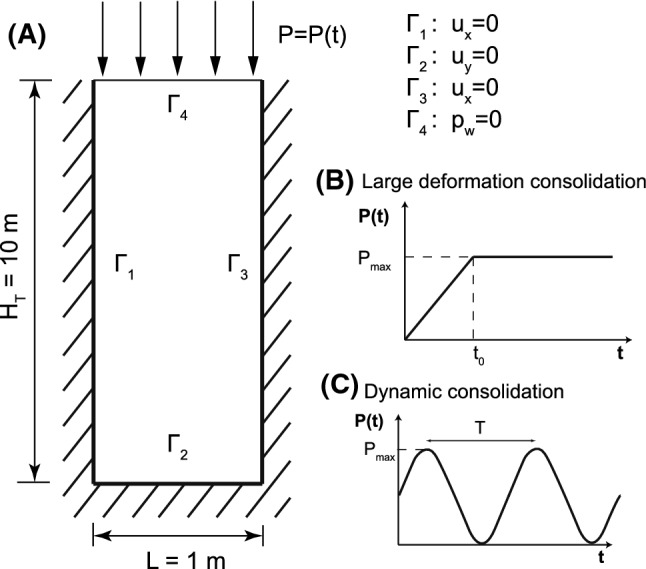
**a** Geometry and boundary conditions of the column of soil; Loading of **b** large deformation consolidation and **c** dynamic consolidation problems

In the following two examples, an idealization of a semi-infinite stratum of soil through a 2D column is employed, which is a traditional procedure seen in the literature. This column has a height $H_\mathrm{T}=10$ m and a width $L=1$ m. Lateral movements are prevented as well as the vertical movement of the rigid base. On the top, the drainage is allowed ($p_\mathrm{w}=0$). This geometry and boundary conditions are depicted in Fig. 1. Also, shape of both loads is depicted for the following problems, large deformation and dynamic consolidations, Sects. 5.1.1 and 5.1.2, respectively.

A regular nodal discretization of 0.5 m. size is employed, taking into account that the last top meter of the stratum is discretized with a 0.25 m. size in order to capture properly the wave provoked by the load. A similar mesh was proposed by Sabetamal et al. [40].

#### Large strain consolidation

Our goal in this section is the verification of the presented methodology when large deformation occurs. Considering this, the consolidation problem solved by Li et al. [23] is performed as a reference. The aforementioned geometry, seen in Fig. 1a, is adopted. The column of soil is loaded following the curve of Fig. 1b; increasing to reach $P_\mathrm{max}$ at $t_0=0.05$ s, when the pressure is kept constant until the end of the simulation (0.5 s). The soild and water parameters are listed in Table 1, being the Neo-Hookean material of Eq. (16) assumed in this case.

**Table 1:** Material parameters of the dynamic consolidation problem

$\lambda$ [MPa]	29	$K_\mathrm{w}$ [MPa]	$2.2 \times 10^4$
*G* [MPa]	7	$K_s$ [MPa]	$10^{34}$
*n*	0.42	$\rho _\mathrm{w}$ [kg/$\text {m}^3$]	1000
*k* [m/s]	0.1	$\rho _s$ [kg/$\text {m}^3$]	2700

**Fig. 2:**
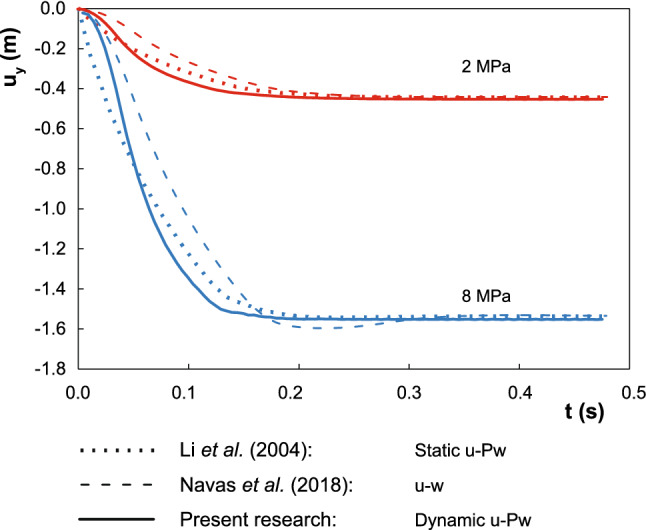
Comparison between the settlement obtained by Li et al. [23], Navas et al. [33] and with the current methodology for the large deformation consolidation problem

The verification is made against the solution proposed by Li [23]. The settlement of the top surface along time is checked for two different values of $P_\mathrm{max}$, namely 2 and 8 MPa, that provide two different scenarios, small and large deformation regimes. The obtained solutions are seen in Fig. 2 for the two cases. Three different solutions are depicted: Static $u-p_\mathrm{w}$ (Li [23]), Dynamic $u-w$ (Navas [33]) and Dynamic $u-p_\mathrm{w}$ (present research). At the final of the consolidation, similar values of the settlement are achieved. Since inertial terms are included in the proposed methodology, the comparison along the entire process described by Li [23] is not possible, since in that research the quasi static $u-p_\mathrm{w}$ formulation is assumed. Consequently, a ramped loading, contrary to the step-wise one employed in [23], is necessary in our case to avoid non-physical sudden loading. Similarly, the results are not comparable against the $u-w$ formulation since fluid acceleration, neglected in the present research, was considered. In addition, the existence of displacements larger than the final settlement between 0.18 and 0.3 s. can be attributed to the above observation regarding the u-w formulation. Since the application of the load is very quick, an impact fluid wave appears, whose propagation is neglected in the proposed formulation.

**Fig. 3:**
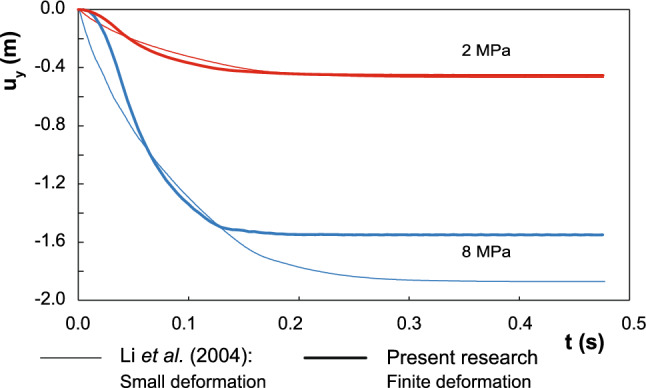
Comparison between the settlement obtained with two different approached: large and small strain regimes

Additionally, the obtained settlement is compared, for both loading states (2 and 8 MPa), against the small strain solution, which was provided also by Li [23]. In Fig. 3, this comparison is plotted. As much is the deformation, as more important is the employment of the Finite Deformation regime since, as it is seen in this application, spurious results can be obtained.

#### Dynamic consolidation

**Fig. 4:**
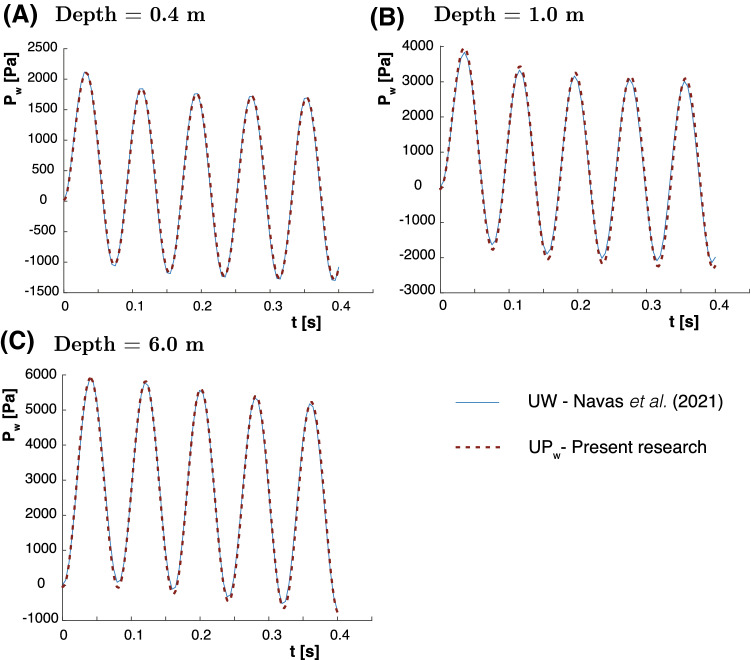
Pore pressure evolution for the Harmonic-Loading ($\omega =25\,\hbox {rad/s}$) consolidation problem at different depths **a** 0.4 m. **b** 1 m. and **c** 6 m

Since soil inertial terms are considered in the proposed $u-p_\mathrm{w}$ formulation, a dynamic problem has been proposed in order to see the performance of the proposed methodology. An interesting test was firstly studied by Sabetamal et al. [40] and later by Monforte et al. [28] and Navas  [32, 35]. Also, the Neo-Hookean material is utilized. The material properties provided in Table 2, and the sinusoidal load, shown in Fig. 1c, are employed. In the aforementioned research, complete formulation ($u-w-p_\mathrm{w}$ and $u-w$ ) results were provided. In this case, $u-p_\mathrm{w}$ solutions of the pore pressure at different locations are presented against the stabilized $u-w$ one in Fig. 4. Slightly differences are encountered. Following, possible reasons are detailed.

**Table 2:** Material parameters of the and harmonic-loading consolidation problem

*E* [MPa]	$\nu$	$K_\mathrm{w}$ [MPa]	$K_s$ [MPa]
30.0	0.20	3.3$\cdot 10^8$	$10^{34}$
*n*	$\kappa$ [m/s]	$\rho _\mathrm{w}$ [kg/$\text {m}^3$]	$\rho _s$ [kg/$\text {m}^3$]
0.33	10$$^{-2} $$	1000	2000

**Fig. 5:**
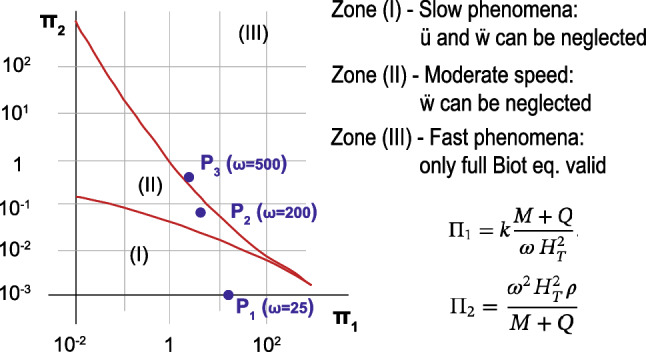
Zones of the different behavior of the soil depending on the parameters $\Pi _1$ and $\Pi _2$ and values of $\omega$ and *k* for the different points to be studied

On the one hand, the differences between the $u-w$ and $u-p_\mathrm{w}$ solutions are small. This is due to the frequency of the load, which is not high enough to provoke water waves and, thus, the acceleration of the water phase can be neglected. Thus, we have to take into account that, following the research of Zienkiewicz and coworkers [53], the configuration of this model lies on the denominated Zone I, where dynamic terms can be neglected (See point 1 of Fig. 5). This is the reason to have similar results for both $u-w$ and $u-p_\mathrm{w}$ formulations.

**Fig. 6:**
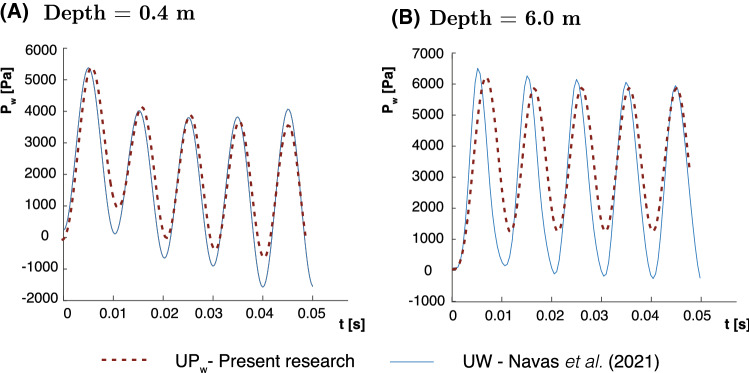
Pore pressure evolution for the medium frequency Harmonic-Loading ($\omega =200\, \hbox {rad/s}$) consolidation problem at different depths **a** 0.4 m. and **b** 6 m

**Fig. 7:**
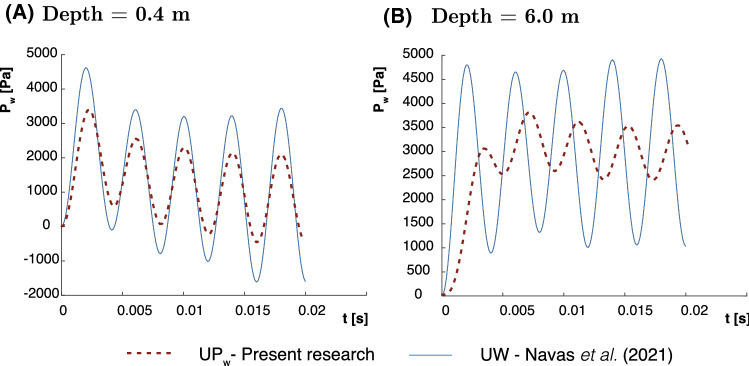
Pore pressure evolution for the high frequency Harmonic-Loading ($\omega =500\,\hbox {rad/s}$) consolidation problem at different depths **a** 0.4 m. and **b** 6 m

Zones of Fig. 5 depend on the geometry, elastic properties, frequency of the load and permeability. By fixing the rest of the parameters and tuning the frequency from 25 to 200 (Point 2 in Fig. 5) and 500 Hz (Point 2 in Fig. 5), our problem becomes Zone II and III, respectively, where dynamic terms are important. Thus, in Figs. 6 and 7, the pore pressure evolution for both approaches is presented for 200 and 500 Hz. It is noticeable the difference, since the $u-p_\mathrm{w}$ is not able to capture several peaks that the $u-w$ does, more displayed for 500 Hz. Indeed, differences are more severe when it is measured deeper in the column, possibly for the undrained behavior. It must be pointed out that, for 200 Hz, no differences should be found. However, the $u-p_\mathrm{w}$ solution is not able to reach $u-w$. Although point 2 is close to the border of Zone III, the figure proposed by Zienkiewicz and coworkers [53] may be updated, at least for the finite strain theory.

**Fig. 8:**
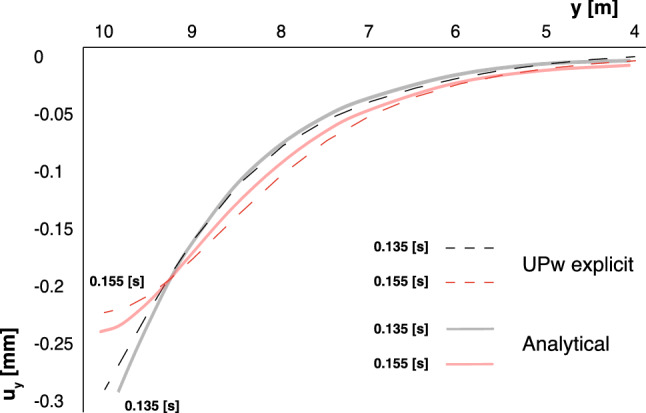
Settlement in the Harmonic-Loading consolidation problem at two different instants: **a** 0.135 s. and **b** 0.155 s

On the other hand, the second comparison is made in the settlement. In Sabetamal et al. [40], we find also the comparison against the analytical solution proposed by De Boer [13], corresponding to incompressible constituents. In Fig. 8, the settlement is plotted for the first 6 m from the top in two instants: 0.135 s. and 0.155 s. There is a slight difference between the peaks.

### Vertical cut

In this Section, the current methodology is applied to the drainage of a square domain of saturated soil loaded on the top right half by a rigid footing. This load provokes the failure of the material in a typical vertical cut, whose shear band varies depending on the material properties, described in Sect. 3 for a hyper-elastoplastic material. Precisely, the importance of this example lies in fact that, depending on the dilatancy angle, the formation of the shear band and the deformation pattern as well as the pore pressure may vary. For all the cases, the friction angle is kept at 20$^\circ$.

**Fig. 9:**
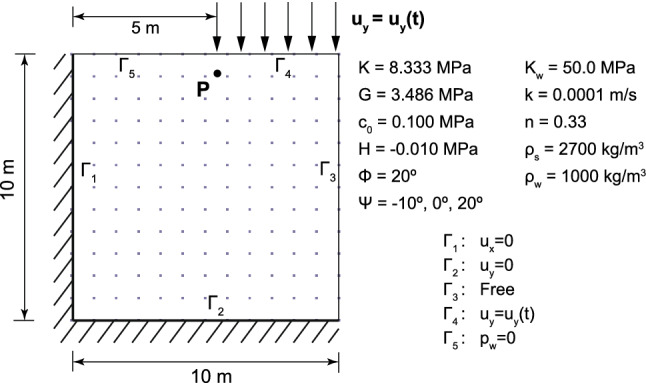
Geometry, material parameters and boundary conditions of a square domain of water saturated porous material

The same problem was previously studied by Sanavia et al. [42, 43] and Navas et al. [33, 34] for both quasi static and dynamic regimes, respectively. The geometry and material properties are shown in Fig. 9. A displacement of 1 m on the loaded boundary, $\Gamma _4$, is imposed gradually during the simulation, which has been fixed of 50 s. A regular 12 $\times$ 12 nodal discretization is employed, which corresponds to a nodal spacing of 0.833 m. The time step is of 5 ms.

**Fig. 10:**
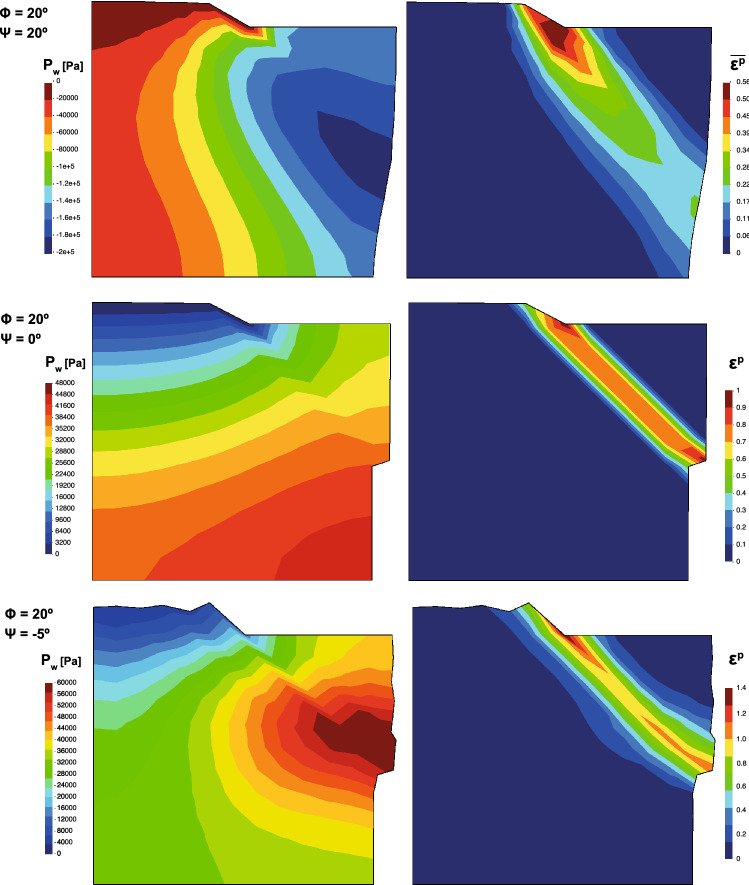
Pore pressure (in Pa) and equivalent plastic strain at 50 s of the square domain for $\psi =20^\circ$, $\psi =0^\circ$ and $\psi =-5^\circ$

Results are depicted, at the final stage, in Fig. 10. In the referred bibliography, we found similar distributions of pore pressure and plastic strain for dilatant, contractive or neutral soils. However, it is worth mentioning that those results were obtained with different coupled formulation, what leads to small difference of the obtained values. Despite this fact, the trend of the behavior of the soil is well captured.

About the shear band, it can be observed that there are no big variations on the obtained peak values of the equivalent plastic strain when the dilatancy angle changes, being slightly bigger when the dilatancy angle decreases. However, an important decrease in the shear band slope is noticed when dilatancy decreases. For associate plasticity, $\psi =20^\circ$, the shear band almost reaches the toe of the lateral wall. It should be noticed that the formation of shear bands induces to locking-based instabilities. Those should be overcome with the appropriate techniques.

**Fig. 11:**
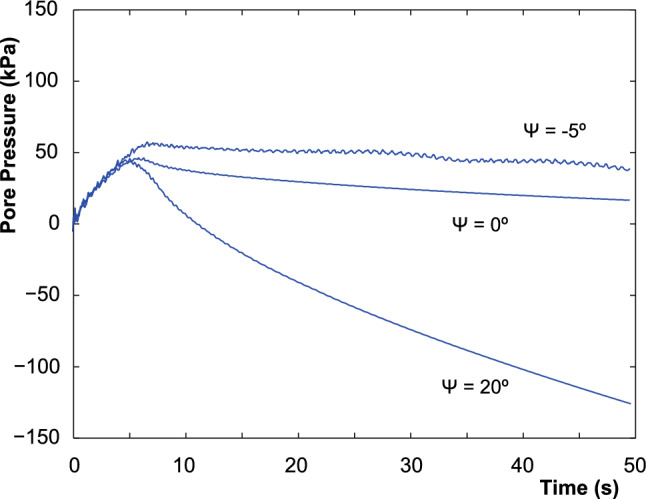
Evolution of the pore pressure along the time in the point P

In addition, the effect of the plastic dilatancy (contractancy) is evidenced by the negative (positive) pore pressure within the shear band zone (see Fig. 10). Moreover, in the case of zero dilatancy angle, no marked pore pressure variation is observed within the shear band zone. Similar phenomena were obtained in the cited research. In order to study the evolution of the principal results of the problem, the history of the pore pressure in a material point close to the shear band (Point P, see Fig. 9) is depicted in Fig. 11.

For positive dilatancy values, smooth pore pressure evolution is observed. In addition, the peak pressure signals the initiation of plastic strain localization or shear band. The further extension of the shear band is accompanied by the continuous decreasing of the pore pressure. The material with dilatancy equal to 0$^\circ$ experiences a softer decreasing (close to a 0$^\circ$ slope), in this case, due to the dissipation of the pore pressure in the permeable boundary, not because of the shear band. From the same figure, it can be seen a very unstable behavior of the soil of contractive angle. This happens since the soil does not admit more load: it is completely failed. In the case of $\psi =20^\circ$, it can be noted in Fig. 11 that the negative water pressure within the shear band is smaller than zero, indicating the possible occurrence of cavitation when lower values of − 98986 Pa (at ambient temperature) are reached. This phenomenon should be modeled by extending the formulation of this work to unsaturated conditions in order to properly capture this phenomenon.

It must be pointed out that, in this research, the sought goal is the assessment of the performance of the proposed algorithm within this geotechnical problem. Other interesting studies of the performance of the Optimal Transportation Method were carried out in [33]. Also, regular distributions provide better results. Finally, the importance of the neighborhood size was assessed, concluding that larger values of $\gamma _{_\mathrm{LME}}$ (which corresponds to smaller neighborhood) reduce the spurious smoothing out of the shear band, being the best results obtained for $\gamma _{_\mathrm{LME}}$=1.4. It is worth mentioning that local processes such as plastic shear band localization are still influenced by the nodal spacing, as well as the achievement of a smoother pore pressure distribution. Following, the influence of the mesh size together with the time step is provided.

#### Study of the discretization size versus time step

**Fig. 12:**
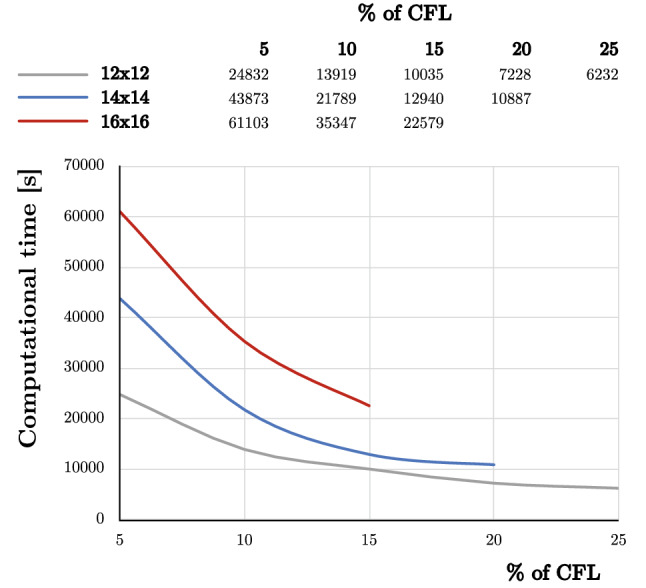
Employed computational time for each type of discretization scheme and different $\Delta t$ as % of CFL

Three different meshes, with different discretization size, have been employed: 12 $\times$ 12 nodes ($h=0.833$ m), 14 $\times$ 14 nodes ($h=0.714$ m), and 16 $\times$ 16 nodes ($h=0.625$ m). For this study, the case with $\psi =0^\circ$ is employed. About the results, according as the mesh gets finer, the maximum percentage of the CFL condition able to be employed becomes smaller. Up to 25% for 12 $\times$ 12, 20% for 14 $\times$ 14 and 15% for 16 $\times$ 16, the simulation becomes unstable. In Fig. 12, the computational costs of all the simulations from 5 of CFL to 25% (when it was possible) are given. It can be observed how the computational time increases exponentially as the time step decreases. Moreover, whether the discretization size gets finer the computational time increases dramatically as well.

**Fig. 13:**
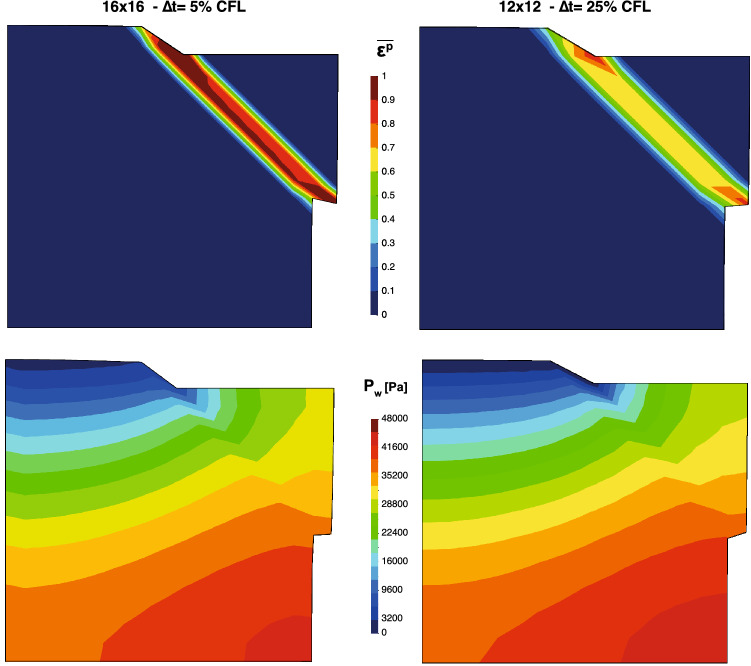
Pore pressure (in Pa) and equivalent plastic strain at 50 s of the square domain for the cases 16x16 and $\Delta t=5\%$ CFL and 12x12 and $\Delta t=25\%$ CFL

Thus, the question of how good is the discretization size for the present problem arises. In Fig. 13, the pore pressure (in Pa) and equivalent plastic strain at 50 s of the square domain for the cases 16x16 and $\Delta t=5\%$ CFL and 12x12 and $\Delta t=25\%$ CFL are depicted, *i.e.*, the largest and the shorter simulations of this study. This comparison gives us the idea of the improvement for a finer spatial discretization and smaller time steps. On the one hand, the pore pressure presents very similar distributions, what let us think that an accurate pore pressure distribution can be captured for relatively coarse discretization nodes at a relatively large time step. On the other hand, a clearer shear band is captured as the nodal size becomes finer. Moreover, since it is more localized, for the same energy, the values the equivalent plastic strain of the finer shear bands are bigger.

**Fig. 14:**
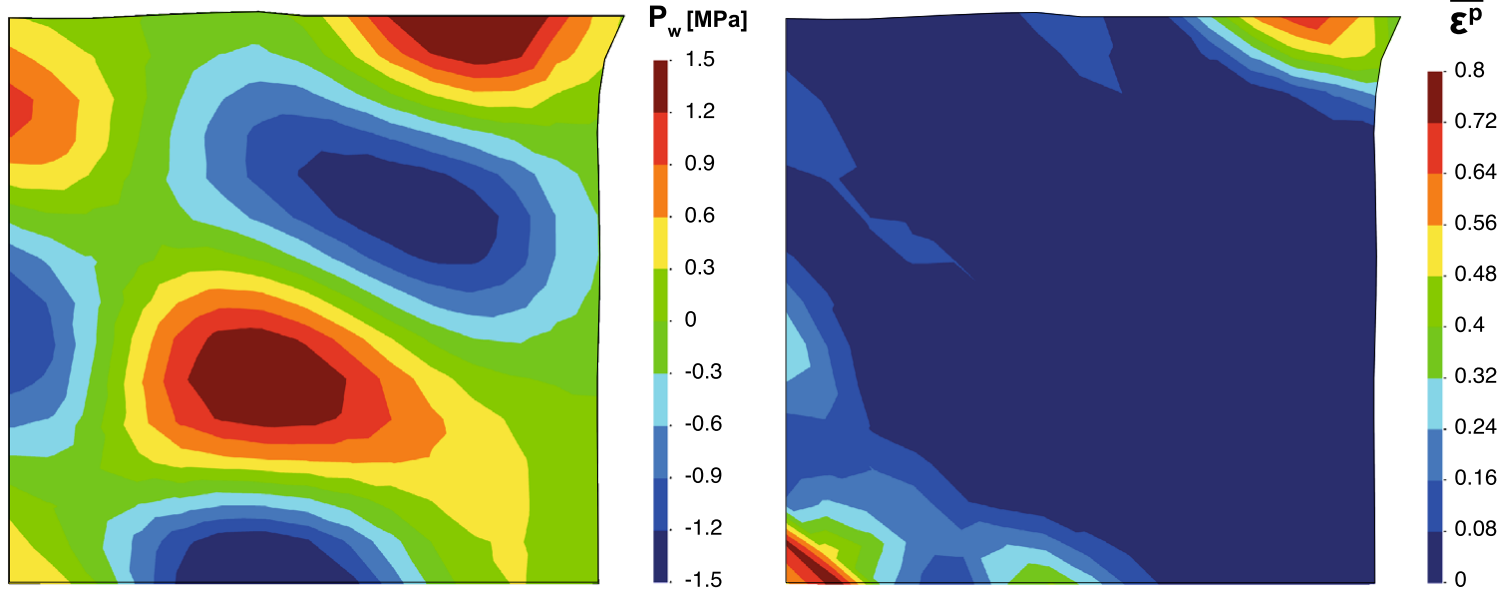
Pore pressure (in MPa) and equivalent plastic strain at 10 s of the square domain for the mesh 12 $\times$ 12 and $\Delta t=30\%\,CFL$

As it was mentioned before, up to 25% for 12 $\times$ 12, 20% for 14 $\times$ 14 and 15% for 16 $\times$ 16, the simulation becomes unstable. It is important to remark this issue as one drawback of the proposed methodology when the material reaches its failure. The simulation behaves stable until the material plastifies. In Fig. 14, the distribution of pore pressure and equivalent plastic strain is seen for the final of the simulation (approximately 10 s.). The pore pressure distribution affects the constitutive behavior, leading to a spurious plastic zone. This pore pressure distribution is the typical one for undrained-incompressible materials: alternate negative-positive values. Although the problem is not in the undrained-incompressible limit, it is necessary the stabilization due to the behavior of the material once the plastification is reached. According with the obtained results of this study, future work will be lead to the stabilization of the proposed formulation.

A remark on the shear band width against the discretization size (Fig. 14) has to be pointed out. These numerical results seem to show a dependency of the shear band width on the discretization size, h. Possible reasons could be due to: (i) the shear band width of the problem is smaller than h, or (ii) the Laplacian term in the mass balance equation is not enough to regularize the strain localization problem upon h refinement. In this case, a regularization procedure could be adopted by modifying the Drucker–Prager criterion adopted in this work (e.g., Perzyna viscoplasticity) as shown in [19].

Finally, as a conclusion of this study, it must be pointed out that, for the appropriate capture of the pore pressure distribution is not necessary to refine the mesh. However, $\Delta t$ in terms of the % of the CFL required to reach stable solutions seems to be relatively low. Moreover, if the problem needs to refine the mesh for any other reason (such as the capture of the shear band), even smaller time steps are required to reach stable results. Thus, parallelization techniques should be employed in order to get accurate results in an adequate computational time. Indeed, further work is required to reach explicit stable solutions for dynamic saturated problems at large deformations within a Max-Ent OTM framework and will be the topic for future work.

## Conclusion

A new methodology to model and compute bi-phase saturated soils at large strains under low/medium frequency loads, by means of an Optimal Transportation Meshfree scheme with an explicit predictor-corrector time integration approach, is proposed.

The robustness of the proposed formulation is assessed by applying it to different well-known geomechanical initial boundary value problems, with both elastic and plastic soil behavior, achieving excellent results. The first example carried out is a consolidation at large strains that was proposed firstly by Li et al. [23]. The behavior of the soil when the range of deformation is big is perfectly captured. In the second example, the model is employed under high frequency loading conditions with a hyperelastic medium. The $u-p_\mathrm{w}$ formulation provides a good performance under low/medium frequency loads, but it is not well suited for high frequency loads. The model is robust and captures both displacement and pore water pressure. Zones of applicability, proposed by Zienkiewicz [55], may be revised in accordance with the results provided in this manuscript. Indeed, the validity when finite strains are employed should be assessed.

Finally, in the last case of analysis, a vertical cut is conducted for a hyper-elastoplastic saturated porous material under a Drucker–Prager flow rule. The proposed model is capable of capturing the complex pore water pressure evolution within the highly distorted plastic shear band in accordance with the dilatancy of the material. Furthermore, the results obtained in the present manuscript are in agreement with the work of Sanavia [43], i.e., contractive materials accumulate pore pressure within the shear band while in the dilatant shear band a reduction in pore pressure is observed.

One of the main conclusions driven by the good performance of the proposed methodology is its extension to other particle-based numerical techniques. Previous research of the same authors, regarding the Material Point Method, shows the excellent fulfillment with Local Max-Ent shape function and an explicit predictor-corrector scheme (see [26, 27]), being both numerical techniques employed within this research. The robustness of the explicit scheme here presented encourages the authors to study other coupled formulations as well as the possibility of making dynamic relaxation techniques in order to extend the range of applicability to long simulations. Even though, since the explicit predictor-corrector time integration approach, considered in the present research, seems to capture adequately the complex hydromechanical behavior at large strains, there are other explicit time integration strategies (Runge–Kutta schemes, embedded Runge–Kutta schemes, symplectic algorithms, Taylor–Galerkin-based techniques, etc. [5, 16, 48]) with already proven capabilities in other scientific fields that should be also considered.

## Nomenclature

- $\varvec{a}^s \equiv \varvec{\ddot{u}}$: acceleration vector of the solid = material time derivative of $\varvec{v}^s$
- $\varvec{a}^{ws}$: relative water acceleration vector with respect to the solid = material time derivative of $\varvec{v}^{ws}$ with respect to the solid
- $\varvec{b}=\varvec{F}\varvec{F}^{T}$: left Cauchy–Green tensor
- $\varvec{\overline{b}}$: body forces vector
- *c*: cohesion (equivalent to the yield stress, $\sigma _Y$)
- $\varvec{C}$ (time integration scheme): damping matrix
- $\frac{D^s\Box }{Dt}$ $\equiv \dot{\Box }$: material time derivative of $\square$ with respect to the solid
- $$\varvec{F}=\frac{\partial \varvec{x}}{\partial \varvec{X}}$$: deformation gradient
- $\varvec{g}$: gravity acceleration vector
- *G*: shear modulus
- *h*: nodal spacing
- *H*: hardening modulus, derivative of the cohesion against time.
- $\varvec{I}$: second-order unit tensor
- $$J=\text{ det }\varvec{F}$$: Jacobian determinant
- *k*: intrinsic permeability
- $$\varvec{k}$$: permeability tensor
- *K*: bulk modulus of the solid skeleton
- $K_s$: bulk modulus of the solid grains
- $K_\mathrm{w}$: bulk modulus of the fluid
- $$\varvec{M}$$ : mass matrix
- *n*: porosity
- $N(\varvec{x})$, $\nabla N(\varvec{x})$: shape function and its derivatives
- *p*: solid pressure
- $$p_\mathrm{w}$$: pore pressure
- $\varvec{P}$ (time integration scheme): external forces vector
- *Q*: volumetric compressibility of the mixture
- $\varvec{R}$: internal forces vector
- $\varvec{s}=\varvec{\sigma }^\mathrm{dev}$: deviatoric stress tensor
- *t*: time
- $\varvec{u}$: displacement vector of the solid
- $\varvec{U}$: displacement vector of the water
- $\varvec{v}^s=\varvec{\dot{u}}$: velocity vector of the solid
- $\varvec{v}^{ws}$: relative velocity vector of the water with respect to the solid
- $\varvec{w}$: relative displacement vector of the water with respect to the solid
- $Z(\varvec{x},\varvec{\lambda })$: denominator of the exponential shape function
- $\alpha _{F}$, $\alpha _{Q}$ and $\beta$: Drucker–Prager parameters
- $\beta$, $\gamma$: time integration schemes parameters
- $\beta _{_\mathrm{LME}}$, $\gamma _{_\mathrm{LME}}$: LME parameters related with the shape of the neighborhood
- $\Delta \gamma$: increment of equivalent plastic strain
- ${\overline{\varepsilon }}^p$: equivalent plastic strain
- $\varvec{\varepsilon }$: small strain tensor
- $\varepsilon _0$: reference plastic strain
- $$\kappa $$: hydraulic conductivity
- $$\lambda $$: Lamé constant
- $\varvec{\lambda }$: minimizer of log$Z(\varvec{x},\varvec{\lambda })$
- $\mu _\mathrm{w}$: viscosity of the water
- $$\nu $$: Poisson’s ratio
- $\rho$: current mixture density
- $$\rho _\mathrm{w}$$: water density
- $\rho _s$: density of the solid particles
- $\varvec{\sigma }$: Cauchy stress tensor
- $\varvec{\sigma '}$: effective Cauchy stress tensor
- $\varvec{\tau }$: Kirchhoff stress tensor
- $\varvec{\tau '}$: effective Kirchhoff stress tensor
- $\Phi$: plastic yield surface
- $$\phi $$: friction angle
- $$\psi $$: dilatancy angle

Superscripts and subscripts

- $^\mathrm{dev}$: superscript for deviatoric part
- $^{e}$: superscript for elastic part
- $_{k}$: subscript for the previous step
- $_{k+1}$: subscript for the current step
- $^{p}$: superscript for plastic part
- $^{s}$: superscript for the solid part
- $^\mathrm{trial}$: superscript for trial state in the plastic calculation
- $^\mathrm{vol}$: superscript for volumetric part
- $^{w}$: superscript for the fluid part relative to the solid one
